# Boosting the mechanical performance and fire resistivity of white ordinary portland cement pastes via biogenic mesoporous silica nanoparticles

**DOI:** 10.1038/s41598-025-86798-y

**Published:** 2025-01-23

**Authors:** Abdallah A. Aziz, Hossam F. Nassar, Mona T. Al-Shemy, O. A. Mohamed

**Affiliations:** 1 Environmental Science and Industrial Development Department, Faculty of Postgraduate Studies for Advanced Sciences, Beni-Suef University, Beni-Suef, 62511 Egypt; 2 Cellulose and Paper Department, National Research Centre, 33El-Bohouth St. (Former El- Tahrir St.), Dokki, P.O. 12622, Giza, Egypt

**Keywords:** Coats-Redfern, Eco-friendly mesoporous silica nanoparticles (MS-NPs), Sustainability, White Ordinary Portland Cement (WOPC), Thermal stability, Environmental sciences, Engineering, Materials science

## Abstract

This study investigates how biogenic mesoporous silica nanoparticles (MS-NPs) extracted from rice straw residues, a sustainable and economical bio-source, affect White Ordinary Portland Cement (WOPC) paste performance. A comprehensive investigation using varied fractions of 0.25, 0.50, 0.75, and 1.0% MS-NPs as an additive to WOPC was conducted to analyze the physicomechanical characteristics of WOPC-MS hardened composites, including compressive strength, fire resistance, and water demand. The beneficial impact of biogenic MS-NPS was verified by X-ray diffraction (XRD), Scanning Electron Microscopy (SEM), and differential thermo-gravimetric analysis (TGA/DTG) methods, revealing several hydration products such as calcium silicate hydrates (CSHs), calcium ferrosilicate hydrates (CFSHs), and calcium aluminosilicate hydrates (CASHs). These products enhance the overall physical and mechanical properties and the thermal stability of hardened WOPC-MS. The composite comprising WOPC-0.75 MS provides numerous advantages from both an economic and environmental perspective.

## Introduction

One of the most ubiquitous building materials in the world is concrete. The composite’s heterogeneity further adds to its complexity. One of the main ingredients in concrete is cement. Cement production is a major polluter because it produces waste products that are both extremely poisonous and destructive^1^. Carbon dioxide (CO_2_), one of these environmental pollutants, is a key exhaust gas in the cement industry and a major global source of CO_2_. Approximately nine hundred kg of CO_2_ equivalent gases are produced for every ton of cement produced, which undoubtedly contributes to additional environmental damage^2–4^. White cement is a specific variety of ordinary Portland cement (OPC). While ordinary cement is frequently employed, white cement offers unique challenges and opportunities for advancement owing to its exceptional brightness and color uniformity. Consequently, selecting white cement enables the development of advanced materials tailored for specific applications that necessitate functional and aesthetic performance, particularly in architectural contexts such as decorative concrete, terrazzo flooring, precast panels, and other scenarios where appearance is paramount. Recycling solid wastes in the white cement manufacturing process was rare due to their high sensitivity to replacement or addition to maintain their whiteness level, chemical and physical properties, etc^5^. In the fresh case, the rheological functionality of cement paste plays an important role in determining the structure of concrete and enhances the mechanical properties and hydration characteristics of hardened white ordinary Portland cement (WOPC)^6^. Therefore, for a more sustainable and eco-friendly option, scientists have investigated the possibility of using agricultural waste ash as a partial cement substitute in concrete mixes^7,8^.

Rice production occupies around 11% of the world’s cultivable land, amounting to 145 million hectares^8^. Rice crop cultivation results in significant amounts of agro-waste, whereas Egypt is estimated to produce approximately 4,968,000 tons of rice straw annually as agricultural waste. Rice waste ash (RWA) can be used as a potential substitute for cementitious material in concrete, either by adding it to or replacing a significant part of the cement in the mixture. It improves the characteristics of both fresh and hardened concrete by promoting the interaction between pozzolanic ingredients and Ca(OH)_2_, leading to the creation of more CSH gel. The procedure described decreases the amount of calcium hydroxide, enhances the compressive strength, decreases the permeability, and enhances the workability of the concrete^9^. Additionally, it mitigates plastic dehydration and shrinkage, boosts the adhesion area between the cement matrix and aggregate, minimizes the permeability of water and chloride, and fortifies the concrete’s resistance to chemical assault. As a result, it reduces the occurrence and advancement of fractures in concrete^8,10^. Therefore, RSA provides substantial advantages in the field of concrete manufacturing.

RWA-cement is less expensive than OPC, as the last one uses limestone, coal resources, and electric power^11^. Furthermore, cement pastes containing RWA produce improvements in mechanical properties^12–14^. The temperature and duration of combustion have a great impact on the RWA’s activity. For example, we can obtain the greatest activity of rice husks by burning it for 2 h at between 600 °C and 800 °C^15,16^. However, the other factors that affect the RWA-cement pastes properties are unknown. The replacement ratios of RWA admixture had a good influence on the cement and concrete performance^17,18^. Considerable research in this area confirms that the ideal replacement ratio of every admixture in various building materials is not the same. RWA includes K_2_O with a high percentage, which may behave as the “activator” through the combustion process. The treatment of rice wastes with acid prior to combustion could manage the removal of these impurities to get an RWA with more and a larger surface area that causes higher activity^19^. Moreover, research showed that the chemical contents of an RWA differ with the source or the origin due to geographical, geological, and climatic conditions^20^.

Nanoindentation has been the tool of choice for numerous studies over the past 20 years to glean useful information regarding the microstructural mechanical characteristics of cementitious composites^21^. Nanoparticles (NPs) are used in a wide range of ways to develop new environmentally friendly technologies for the industry of cement, which is the economical experience and the leading of the hour. Significantly, these novel procedures possess the capacity to reduce environmental pollution as compared to traditional manufacturing methods substantially^22–27^.

Nano-silica (NS), as filler, is a highly effective pozzolanic material. They are very fine and glassy particles, about a thousand times smaller than the average cement particles. It forms a premium mixture with cement to make the quality of the cementitious complex or compounds get better to a great extent. Cement pastes containing NS recorded rapid and accelerated hydration; this may be due to the chemical reactivity (pozzolanic reaction) or high surface area^28^. The pozzolanic reaction causes a reduction in the amount of calcium hydroxide in the concrete mixture and reduces the hydration time, water absorption, porosity, and permeability. According to earlier studies, adding NS improves the properties of both the fresh and hardened material more than adding other mineral additions. Using NS with cement pastes improves the cohesive force of the entire mixture in its fresh condition, lowers the amount of water needed, and shortens setting periods compared to other silica compounds like silica fume. Because particle agglomeration during mixing causes problems, scientists and authors estimate that the ideal effective proportion of NS should be less than 5% by weight. According to some scientists, the right ratio is 10% by weight, depending on the modifications that must be made to the components to prevent excessive self-drying and strength impairment from microcracks^29,30^. Adding or replacing a part of the cementitious materials of WC with NS focuses on developing its unique characteristics and reduces its manufacturing cost and environmental drawbacks^31^.

The mesoporous silica’s nanoparticle (MS-NPs) surface measures over 900 g/m^2^ ^32^. The influence of NS with mesoporous MCM-41 is reviewed on plain concrete’s compressive^33^. Previous research examined the impact of the method of incorporating MS-NPs into cement mortar. It was found that adding these nanospheres to an acetone solution along with cement works better and gives the concrete higher compressive and flexural strengths than putting them into a water solution with cement^34^. For all these reasons, a green environment can be obtained besides enhancing the properties of WOPC. Consequently, in order for the green environment to accomplish its sustainability goals, it was necessary to seek out new methods and create them in order to recycle a substantial quantity of rice waste. The new process of recycling agricultural waste allows for the evaluation of WOPC pastes’ qualities without compromising their sensitivity.

The purpose of this research is to examine the physico-chemical properties of WOPC that contain mesoporous silica nanoparticles (MS-NPs). So, different MS-NP ratios were tested to see how they affected the mechanical and hydration features of hardened WOPC. For every cement paste, its water consistency was recorded, and its first and last setting times were measured. In addition, up to a 28-day curing period, the compressive strength, chemically mixed water, and free lime values of the hardened cement specimens were recorded. Certain hardened samples were examined using XRD, TGA/DTG, and SEM techniques to track the hydration rate of the prepared materials.

## Experimental program

### Resources of materials and their characterizations

The rice straws were from Delta, Egypt, which has a moderate temperature, sunny climate, and sufficient humidity. The chemical composition of the rice straw residues was established using TAPPI standard procedures. The results showed the following: silica 14% (T 245 om-94), ash 15.09% (T-211), lignin 15.09% (T-222 om-88), extractives 3.95% (T 204 cm-97), hemicellulose 21.35% (T-223 cm-84), and α-cellulose 42.12% (T 203 cm-99). WOPC (52.5 grade, Blaine specific surface 448 m^2^ /kg, containing 5% limestone powder) is used in this study. The whole chemical analysis of WOPC was performed by X-ray fluorescence (XRF: Xios, style PW-1400) and the chemical composition of WOPC was determined and tabulated in Table 1.

**Table 1:** Chemical oxide compositions of WOPC.

SiO	Components	Cl	SO3	K2O	MgO	Na2O	CaO	Fe2O3	Al2O3
23.3	WOPC	0.04	3.43	0.06	0.29	0.12	67.2	0.15	2.38

### Fabrication of eco-friendly mesoporous silica nanoparticles (MS-NPs)

In this study, SiO_2_NPs with controlled shape, phase, and purity were produced using rice straw residues as a natural and affordable bio-source. The procedure used to accomplish this was as follows: The milled RS sample was first leached with 0.5 M HCL at a consistency of 10% (wt/v) and 80 °C for 30 min. The resulting slurry was then filtered, cleaned with tap water, and allowed to dry at 50 °C overnight. Leached RS was calcined twice, the first time at 350 °C for 45 min and the second time at 700 °C for an additional 180 min. After that, silica was extracted from RS ash by dissolving it in 2 M sodium hydroxide (10% wt/v) at 100 °C for four hours (Eq. 1) while vigorously stirring the mixture in a closed system. To get rid of carbon leftovers that hadn’t been burned, the resulting sodium silicate solution was filtered. Following its neutralization with a 2 M hydrochloric acid aqueous solution, it underwent another precipitation (Eq. 2) ^35^.

1 $$2NaOH{\text{ }}\left( {aq} \right)\,+\,2Si{O_2}\left( s \right){\text{ }} \to {\text{ }}N{a_2}Si{O_3}\left( {aq} \right)\,+\,{H_2}O$$

2 $$N{a_2}Si{O_3}\left( {aq} \right)\,+\,2HCl{\text{ }} \to {\text{ }}Si{O_2}\left( {gel} \right) \downarrow {\text{ }}+{\text{ }}2NaCl{\text{ }}\left( {aq} \right)\,+\,{H_2}O{\text{ }}\left( l \right)$$

The reaction between sodium silicate and hydrochloric acid facilitates the generation and merging of silanol groups (R_3_Si-OH), creating a three-dimensional Si-O-Si network^36,37^. Silica ash produced was 140 g per 1000 g of rice straw residue (14% yields). Treating it with alkali using the previously indicated process formed 100 g of MS-NPs (71.5% yields). The products were then evaluated utilizing a range of analytical tools, such as XRD, EDX, FTIR, TEM, TG, and N_2_ physisorption isotherm studies.

### Cement paste composite preparation and testing protocols

WOPC was replaced by 0, 0.25, 0.50, 0.75, and 1.0% of MS-NPs, as shown in Table 2. The water-to-cement ratio (W/C ratio) was adjusted according to the weight% of MS-NPs combined with cement pastes. To avoid MS-NP coagulation, pre-dispersion of MS-NPs is necessary prior to cement mixing. Therefore, the MS-NPs are dissolved in the entire mixing water, then dispersed by using the ultra-sonication technique for 20 min, after which the emulsion distributed in a multi-speed blender was mixed with cement to form cement paste, followed by pouring the pastes into cubic molds having the following dimensions of 2.5 × 2.5 × 2.5 cm^3^, then kept at high relative humidity (nearly 100% RH) for 24 h. After that, the samples were demolding and immersed under tap water at normal temperature for 28 days of curing time^38^.

**Table 2:** Optimum water of consistency and setting time for the WOPC-MS hardened composites.

Mixes	Mix proportion (Wt, %)		W/C (%)	Initial setting time (min)	Final setting time (min)
	WOPC	MS-NP			
Mix WMS0	100	-	0.29	125	180
Mix WMS1	100	0.25	0.292	95	140
Mix WMS2	100	0.5	0.293	105	150
Mix WMS3	100	0.75	0.295	115	160
Mix WMS4	100	1	0.296	125	175

In accordance with industry standards (ES 2421-3; UNI EN 196-3), setting time and standard water consistency for these pastes were examined. Using a VICAT device to measure the setting time^39^. According a ASTM C109M (2016), the compressive strength (CS) was determined by using Ton industrie instrument (West Germany) at different curing time intervals of 1, 3, 7, and 28 days^40^. The hydration reaction was stopped using stopping (1:1 acetone - methanol) mixture with stirring for 1 h, then filtered and dried at 75 °C for 3 h. After that, the stopped samples were maintained in desiccators^41^.

By heating the ground dried specimens at a temperature of 105 °C to remove any free water to prepare the sample for measuring the chemically combined water content, then for one hour at 1000 °C, the chemically combined water content (Wn, %) was calculated from the ignition loss. For every specimen, duplicate measurements were made, and the average value was noted. Wn, %= [(W_0_ – W_f_) / W_f_] × 100; where W_0_ = dried specimens mass and W_f_ =ignited specimens mass.

Chemically combined water (Wn, %) and free lime (CaO, %) contents were calculated^42^.

After 28 days of curing time, a fire resistance test was performed in which the hardened composite pastes were maintained at approximately 75–80 °C for 1 day and then exposed to firing at 250, 500 and 750 °C for 3 h. The cooling process was applied to the fired samples in Two different ways (gradually and rapidly cooling)^43^.

### Characterization

A JEOL JEM-2100 transmission electron microscopy (TEM) system was used to conduct high-resolution TEM investigations on the produced MS-NPs. The elemental analysis of the extracted MS-NPs was conducted using an Energy Dispersive X-ray (EDX) spectrometer on a Quanta FEG-250 microscope, operating at a voltage of 10 kV.

The surface area and pore size of the MS-NPs were assessed using a Quantachrome (USA; Nova 2000 series) device employing the Brunauer-Emmett-Teller (BET) model. The samples were subjected to overnight heating at a temperature of 150 °C in order to remove any trapped gases. The physisorption isotherm at 77 K was conducted using nitrogen gas. The FT-IR spectrum of the MS-NPs was recorded using a JASCO FTIR 6100 spectrometer. The spectrometer had a resolution of 4 cm^− 1^ and a range of 4000 –400 cm^− 1^, and 60 scans were performed.

The phase purity, composition, and crystallinity of the different produced samples were determined by X-ray powder diffraction (XRD) in the 2θ range of 5°–80°. The Panalytical Empyrean X-ray diffractometer was employed for this purpose.

The cement and the MS-NPS samples that were made were both tested for thermal degradation using a differential scanning calorimeter (SDTQ600 V20.9 Build 20). The measurements were taken in an inert nitrogen environment at a testing rate of 20 °C/minute, with temperatures ranging from 25 to 800 °C, in order to avoid degradation occurring too soon. Equation 3 was utilized in conjunction with the Coats-Redfern approach to ascertain the energy of activation (E^a^)^44^:

3 $$\log \left\lfloor {\frac{{\log {W_f}/({W_f} - W)}}{{{T^2}}}} \right\rfloor =\log \left[ {\frac{{AR}}{{\theta {E_a}}}\left( {1 - \frac{{2RT}}{{{E_a}}}} \right)} \right] - \frac{{{E_a}}}{{2.303RT}}$$

Here, R is the gas constant, θ is the heating rate, and W and Wf are the sample’s actual and final weights (in grams) up to temperature T (in Kelvin). Equation (4) were used to compute the additional kinetic parameters: The free energy change of activation (ΔG_a_), the entropy of activation (ΔS_a_), and the enthalpy of activation (ΔH_a_)^45^.

4 $$\:{\Delta\:}{\text{H}}_{a}=\:{\text{E}}_{a}-\text{R}\text{T};\:{\Delta\:}{\text{S}}_{a}=\:2.303\text{R}\left(\text{l}\text{o}\text{g}\frac{\text{A}\text{C}}{{\text{K}}_{b}\text{T}}\right),\text{a}\text{n}\text{d}\:{\Delta\:}{\text{G}}_{\text{a}}={\Delta\:}{\text{H}}_{a}-\text{T}{\Delta\:}{\text{S}}_{a}$$

Here, *K*_*b*_ and *C* are Boltzmann and Planck constants, respectively.

## Results and discussion

### Extraction and characterization of MS-NPs

**Fig. 1:**
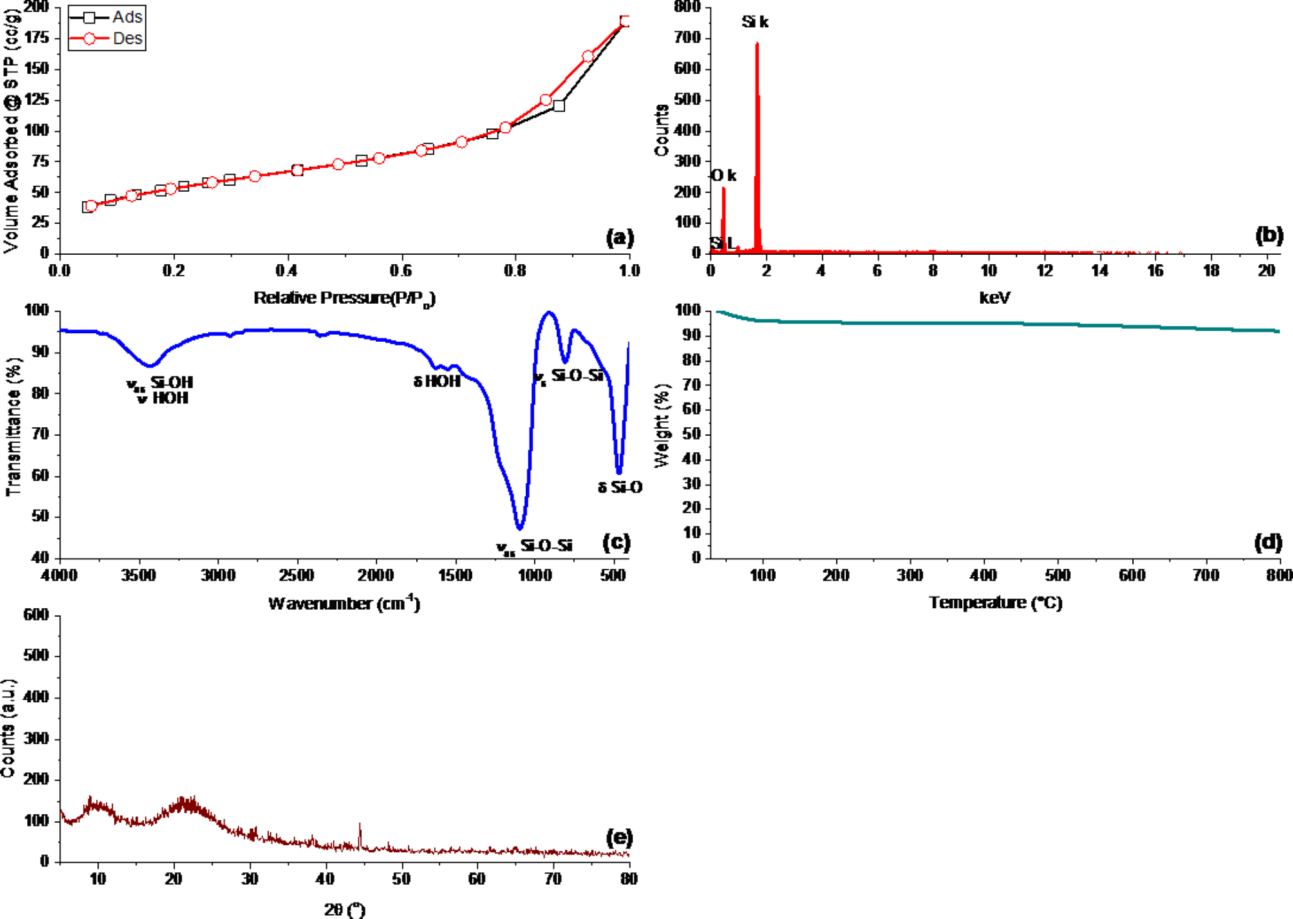
(**a**) N_2_-adsorption/desorption isotherm, (**b**) EDX analysis, (**c**) FTIR spectra, (**d**) TGA thermograms, and (**e**) XRD patterns MS-NPs.

The N_2_ physisorption isotherm of the manufactured MS-NPs sample is depicted in Fig. 1a. Physisorption pores can be conveniently classified based on their size, following the guidelines of the International Union of Pure and Applied Chemists (IUPAC). Micropores are defined as pores with a width of 2 nm or less, mesopores are pores with a width between 2 nm and 50 nm, and macropores are pores with a diameter of 50 nm or greater^46^. Given that the average pore width of the manufactured SiO_2_NP sample is more than 6 nm, it can be classified as a mesoporous material. The mesoporous materials’ type IV isotherm was visible in the produced MS-NPs^47^. MS-NPs’ adsorption and desorption assays revealed significant BET surface areas (187 m^2^/g), total pore volumes (0.29 cm^3^/g), and average pore diameters (6.14 nm), all of which were caused by the presence of inter-particulate holes between the NPs. The N_2_ adsorption-desorption isotherm showed an H3 hysteresis loop because there was no saturation adsorption at a high relative pressure (P/P_0_). This was because the particles were arranged in a flexible way, like lamina^46,48^. This hysteresis loop structure in fabricated MS-NPs is a consequence of capillary condensation with hysteresis, which occurs with mesoporous sorbents with an average pore size of more than 4 nm and is controlled by the sorbent texture, according to the IUPAC^44,47^.

By using EDX analysis, the produced MS-NPs’ purity was identified. According to Fig. 1b, the only components present in the MS-NPs produced were silica and oxygen.

The synthesized MS-NPs used in this study have a molecular structure that is discernible from FTIR analysis, as shown in Fig. 1c. The isolated MS-NPs’ vibrational bands at about 3436, 2921, 1627, 1552, 1095, 806, and 470 cm^− 1^ included adsorbed moisture and/or amorphous silica and/or silicic acid^37,49^. Si-OH showed symmetric and asymmetric vibrational absorption bands at 3436 cm^− 1^ and 806 cm^− 1^, respectively^50,51^. The estimated adsorbed moisture of 4.38 wt% for MS-NPs, as determined by TG analysis, may be the reason for the broadening of the silanol peak between 3070 and 3750 cm^− 1^ owing to the H-bonded silanol groups. At around 1627 and 3436 cm^− 1^, respectively, the bending and stretching vibrational peaks related to adsorbed moisture occurred^52^. The Si-O-Si groups from the silicate matrix also showed symmetric and asymmetric vibrational absorption bands at around 1095 and 806 cm^− 1^, respectively. Along with bending vibration at around 470 cm^[− 1 53,54^.

Additionally, TG analysis was carried out to assess the purity of isolated MS-NPs (Fig. 1d). In the temperature range of 40–150 °C, TGA thermograms for as-produced MS-NPs only show one dominant pyrolysis phase with weight loss corresponding to 4.38%. Volatilization of the adsorbed water molecules and complete siloxane linkage conversion of the remaining silanol groups cause this process^55^. Another indication that pure mesoporous MS-NPs were completely manufactured is their thermal stability following this stage of weight reduction^56^.

MS-NPs were subjected to an XRD examination to show whether the materials were crystalline or amorphous. According to Fig. 1e, the major diffraction peak in the retrieved MS-NPs’ diffraction profiles is indicative of the amorphous phase of mesoporous MS-NPs that occurs at a Bragg angle of 22°^54^. As a result, the XRD profile shows that the amorphous silica was successfully extracted.

**Fig. 2:**
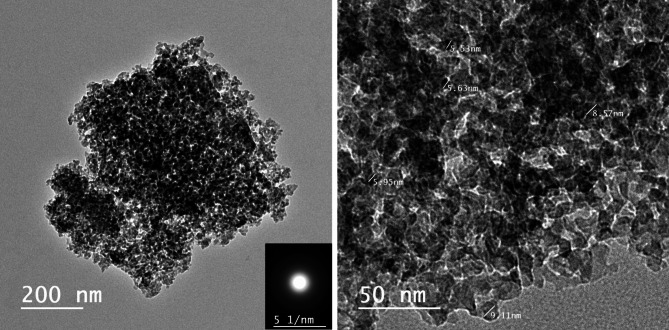
HR-TEM of MS-NPs.

Due to their consistent mesoporosity, large surface area and pore size, particle size (10–1000 nm), tunable pore diameter (2–30 nm), surface functionalization, flexible morphology, superior biodegradability, and biocompatibility, MS-NPs have attracted a lot of attention^57–59^. Using TEM, the shape of the produced mesoporous MS-NPs derived from burned RS was investigated. Figure 2: Single uneven chunks with a width varying from 160 to 900 nm are visible in TEM micrographs. These masses of MS-NPs are homogeneous, spherical, and somewhat regular in their holes and voids, which range in diameter from 9.11 to 5.53 nm, much like mesoporous structures. In line with the N_2_ physisorption isotherm data, the TEM data show that the mesoporous MS-NPs that were successfully made were in fact periodic matrices consisting of medium-sized pores contained inside an amorphous silica matrix.

### Physicomechanical aspects

#### Setting characteristics of white cement paste

**Fig. 3:**
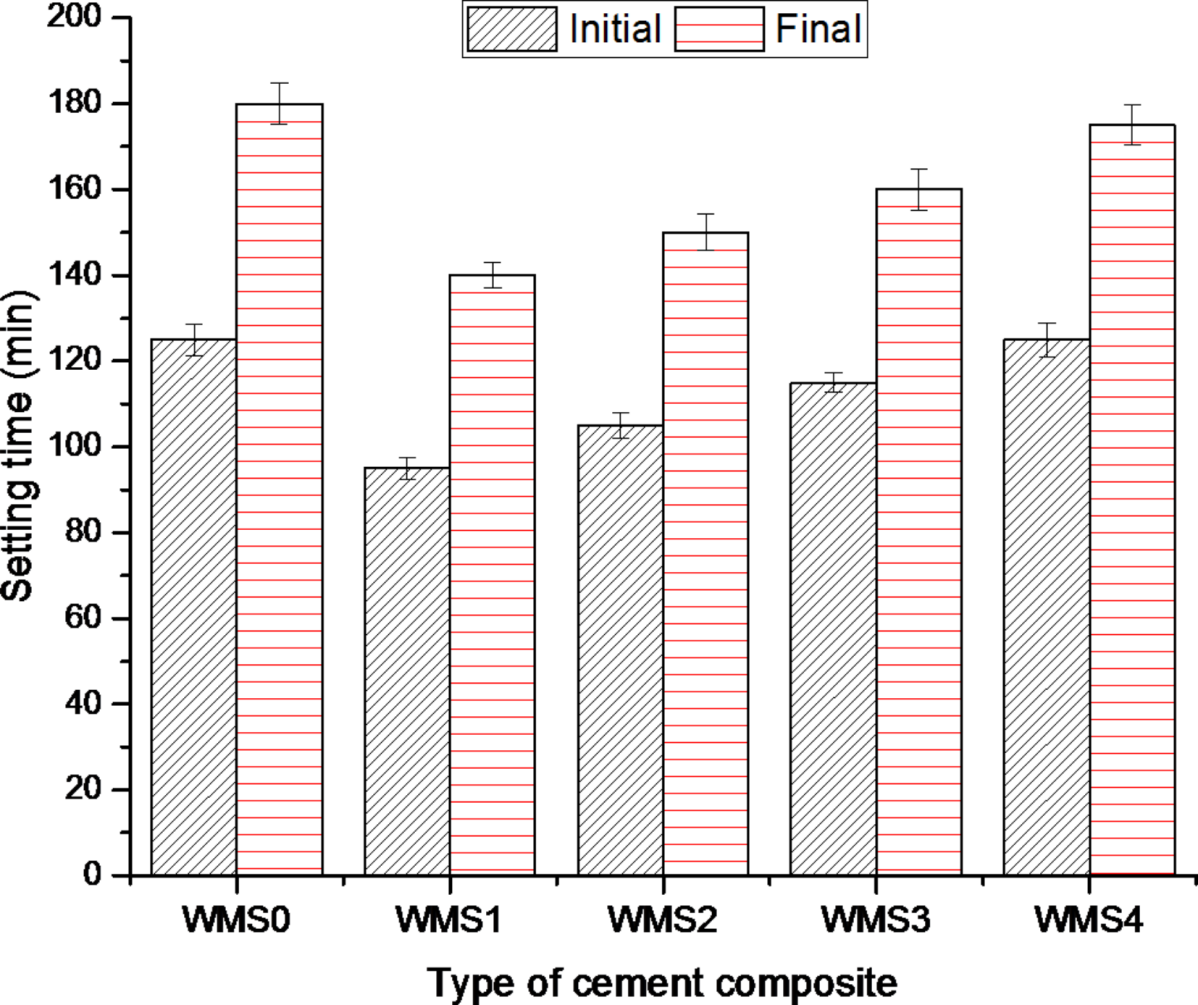
Setting times of the neat WOPC (Mix WMS0) and WOPC-MS composites.

Standard water of consistency and Setting time for the WOPC-MS hardened composites are shown in Table 2; Fig. 3, shows the both initial and final setting times of WOPC-MS hardened composites as a function of time. It can be observed that the values of standard water consistency of all admixed cement pastes are slightly higher than that of WOPC blank (Mix WM0), as more water consistency is required in order to high dispersion of the MS, which causes an increasing in w/c ratio and speeding up the rate of hydration of cement^60^. Also, the obtained results indicate that the addition of MS for cement pastes results in a notable reduction in time taken to set, so that all setting time values obtained for WOPC-MS hardened composites are shorter than those of the blank Mix (WM0). The shortest setting time behavior of MS may be attributed to the nucleation effect, the combination of pozzolanic activity, and the filling effect of MS during the hydration process^38,61,62^. It was noted that as the amount of addition of MS increases, both the initial and final setting times increase owing to the compactness and accumulation of the small nanoparticle of MS, which would have reduced the penetration rate of water and the rate of hydration reaction, and consequently increased the setting time. Mix (WM1) has the quickest setting times when compared to other WOPC composite cement blends containing MS where MS aids as an active crystal site for the generation of an excess of calcium aluminate hydrate (CAH), calcium silicate hydrate (CSH), and gel calcium aluminosilicate (CASH) and further CSH stages subsequence on.

#### Compressive strength (CS)

**Fig. 4:**
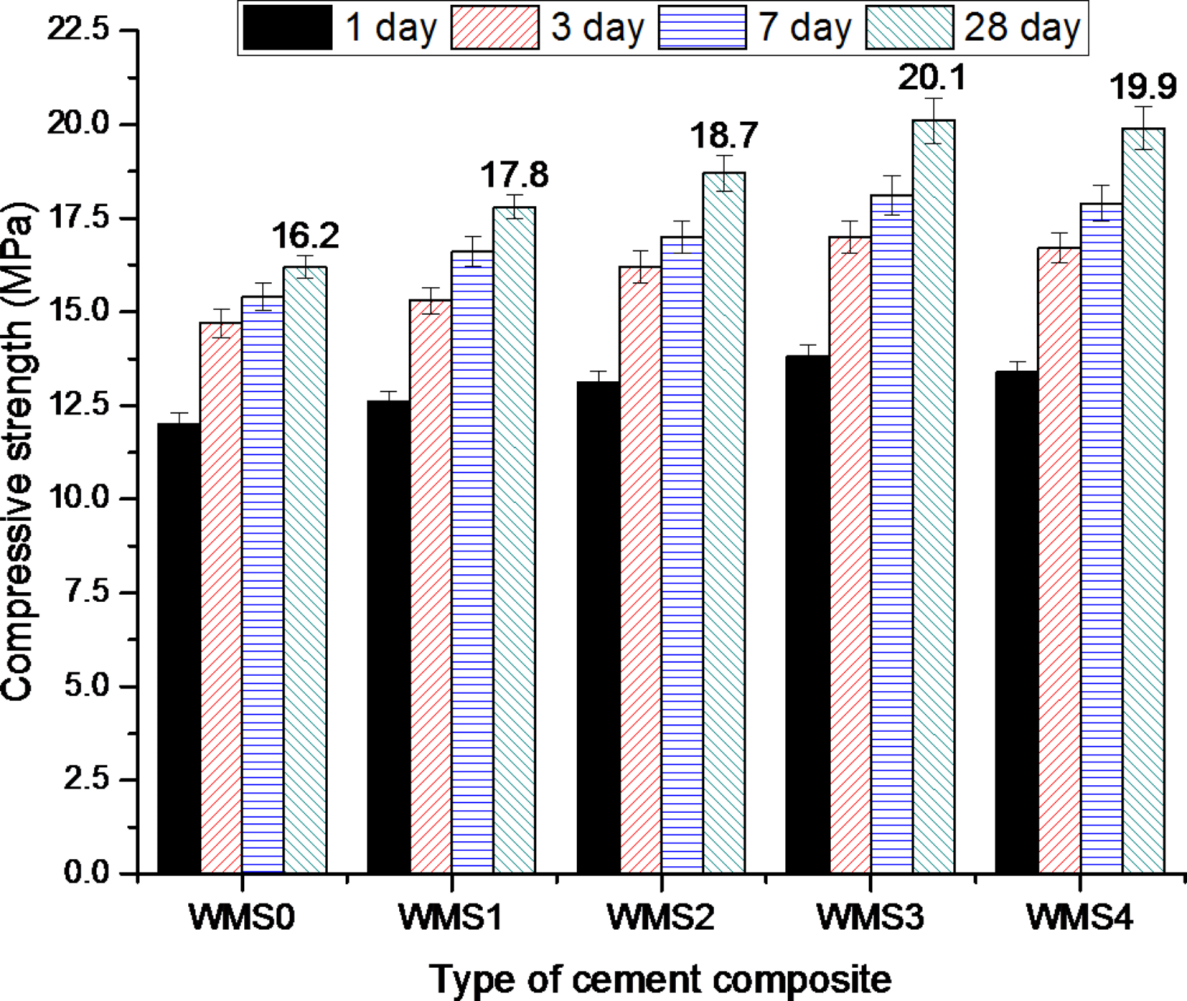
Compressive strength values for hardened composites made from WOPC-MS at different ages of hydration.

Figure 4 represents the results of the CS values of the hardened neat WOPC pastes, as well as WOPC pastes modified with various additions (0.25, 0.50, 0.75 and 1%) of (MS) of 6.14 nm average particle size. As can be seen clearly from Fig. 4, the values of compressive mechanical strength showed a continuous overall development with the increase of the hydration time for all hardened MS-white ordinary Portland cement pastes. In general, the hydration of different phases in WOPC clinker and the subsequent formation of hydration products such as ettringite (C_6_AŠ_3_H_32_), calcium aluminosilicatehydrate (C(A)SH), alumino ferrrite monosulfate hydrate (AFm), calcium aluminate hydrates (CAH), and calcium silicate hydrates (CSH) are the main causes of the ongoing rise in strength values. Adding MS improved the strength of the WOPC-MS hardened composites. MS significantly influences the early hydration process (up to 7 days) by enhancing the matrix structure of the WOPC-MS-hardened composites. Additionally, it contributes to a slower increase in CS values later on by protecting the un-hydrated parts of cement grains with the initial hydration products, leading to a gradual rate of hydration^42^. The WOPC-MS hardened composites with 0.25%, 0.50%, 0.75%, and 1.0% of MS-NPs displayed a similar trend to the blank (Mix WMS0), but exhibited significantly higher values of CS, particularly in the initial stages of hydration (up to 7 days). It is evident that the WMS3 mix, which contains 0.75% MS, demonstrated the highest strength value after 28 days compared to the other mixes (WMS0, WMS1, WMS2, and WMS4). The WOPC-MS composites were strengthened with varying percentages of MS-NPs: 0.25%, 0.50%, 0.75%, and 1.0%. This improvement comes from the filling effect of integrated mesoporous silica (MS), and the cement matrix becomes less porous. Furthermore, the results indicated that the extent of reduction in the CS values was heightened as the percentage of MS added to WOPC rose. This could disrupt the composite microstructure.

### Cement pastes hydration

#### Chemically combined water content (wn)

**Fig. 5:**
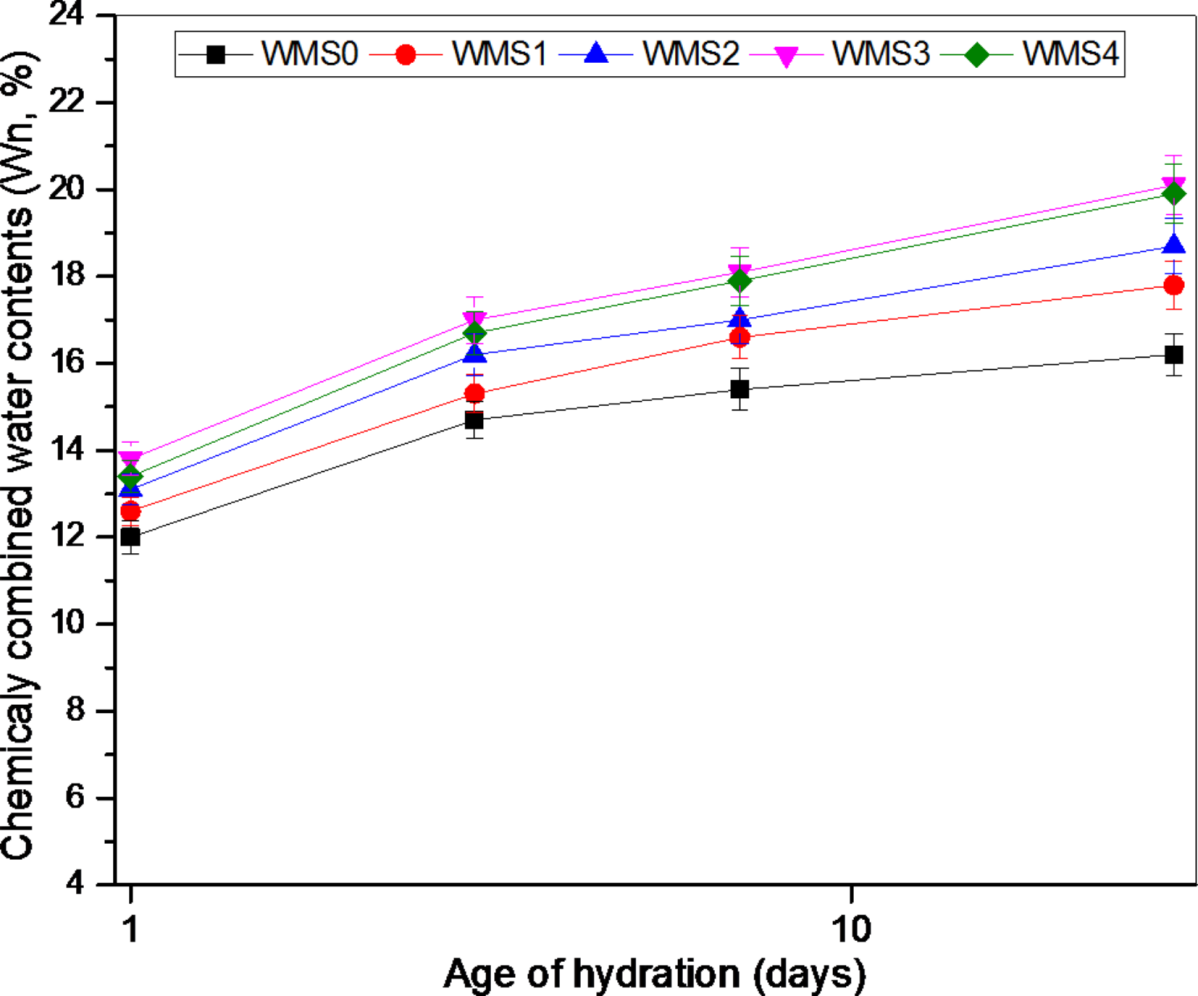
Chemically combined water contents for hardened composites made from WOPC-MS at different ages of hydration.

Figure 5 demonstrate the results of Wn for the WOPC-MS hardened composites pastes. Evidently, the Wn - values obtained for all the tested hardened samples indicate a gradual continuous increase up to the final hydration ages studied (28 days). The Wn values obtained for all the evaluated hardened samples demonstrate a steady and continuous rise until the ultimate hydration age is examined (28 days). The observed outcome is attributed to the advancement of the hydration process and the spread of calcium silicate hydrates (CSH) and calcium hydroxide (Ca(OH)_2_) as essential byproducts of hydration^63^. The cement pastes composed of Mixes (WMS1 – WMS4) exhibited significantly greater values of Wn compared to plain WOPC at all hydration stages. Furthermore, Mix WMS3 (containing 0.75% MS) resulted in the greatest Wn values among all tested specimens after 28 days compared to those of the other mixes (WMS0, WMS1, WMS2 and WMS4). This finding is owing to the advancement effect of MS resulting in acceleration of cement hydration process with a stronger and denser microstructure via the generation of further hydration products like C(A)SH, C-A-H, and C-S-H that deposit in the pore structure.

#### Free lime content (FL)

**Fig. 6:**
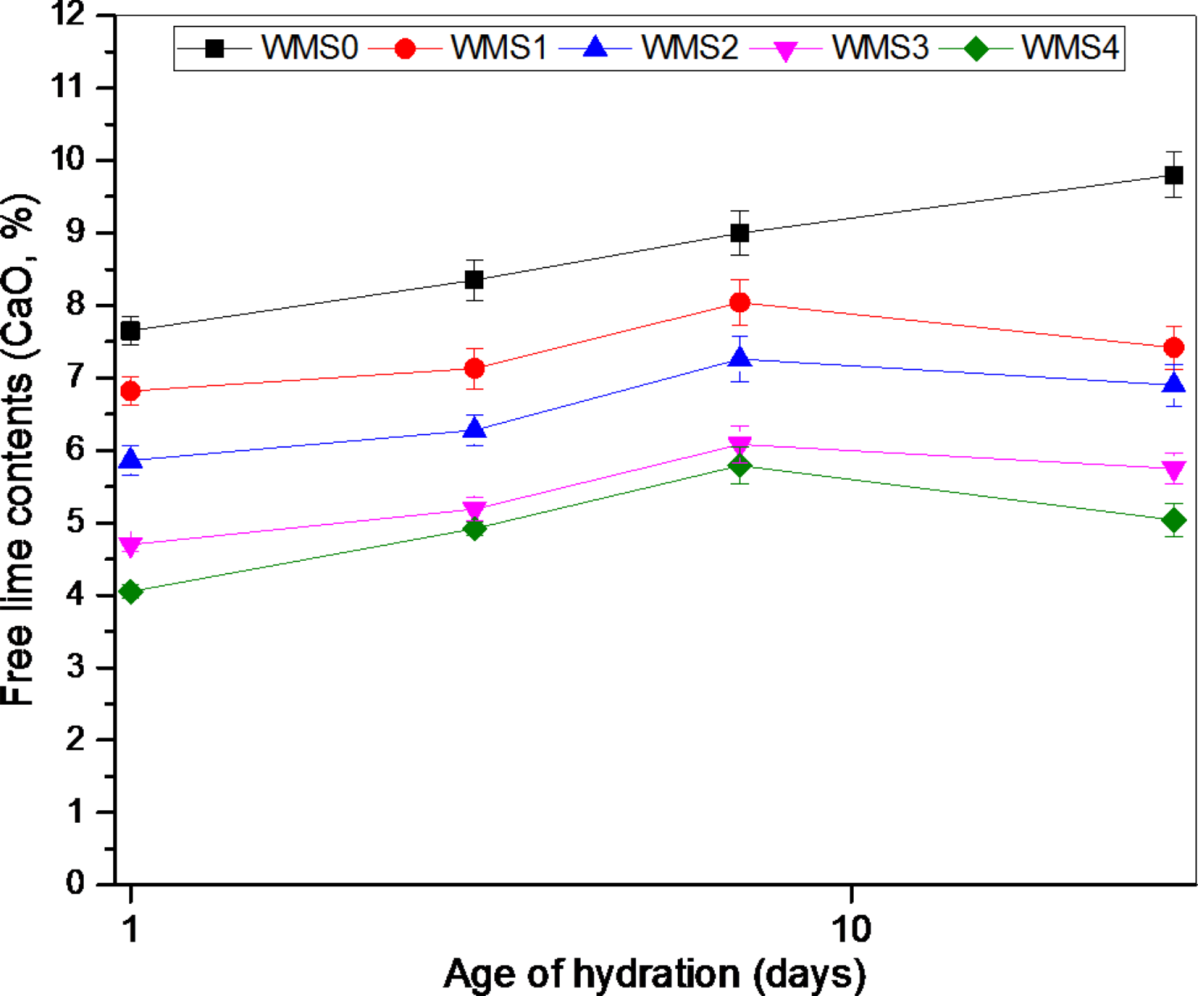
Free lime contents of neat WOPC (Mix WMS0) and WOPC-MS hardened composites at different ages of hydration.

Figure 6 presents FL content (CaO, %) for neat WOPC (mix WMS0) and WOPC-MS pastes modified with various amounts of MS (WMS1, WMS2, WMS3, and WMS4 composites) cured up to 28 days. The free lime content (FL) increases with increasing curing age; this may be owing to the progress of the hydration reaction, which liberates free portlandite CH through the hydration time^64^. The findings also demonstrate a reduction in the values of the free lime content (FL %) for the WOPC pastes containing MS nanoparticles during all ages of hydration; and the reduction in FL contents (CaO, %) is notable with increasing the % of MS. There are two opposite processes for all pastes containing MS, the first one is a slight increase in FL content (CaO, %) due to liberated free portlandite from cement hydration up to 7 days and the second process is a marked decrease in FL content (CaO, %) up to 28 days. This decrease in (FL %) values is attributed to MS nanoparticle’s pozzolanic activity; hence MS can minimize Ca(OH)_2_ and aid the forming of a denser system containing C-F-H and C-F-S-H gels, besides further amount of C-S-H.

### Thermal degradation (TD)

**Fig. 7:**
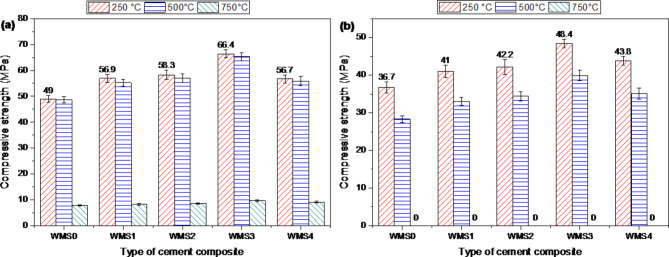
Compressive strength values for hardened composites made from WOPC-MS fired at different temperatures and cooled (**a**) gradually in air, (**b**) suddenly in water.

Figure 7a presents the influence of higher temperatures (250, 500, and 750 °C) for 3 h. Then, allow them to cool gradually in the ambient air, changing the CS values for plain WOPC and WOPC-MS composite cement pastes. The data in the figure shows a significant increase along thermal treatment upon heating at 250 °C in CS values for all fired composites compared to their recorded values after 28 days of hydration, followed by a slight decrease upon heating at 500 °C, then a sharp reduction for all composites up to 750 °C. Really, the severe boost in CS after firing at 250 °C could be attributed to the internal (self-autoclaving) process created from the elimination of the physically adhered water molecules in cement structure. Composite pastes, WOPC-MS, were enhanced with several amounts (0.25, 0.5, 0.75, and 1 mass %) of MS-NPs. These modified pastes exhibited higher CS values after being fired at 250 °C compared to the CS values of the plain composite (Mix WMS0). After being fired to 250, 500, and 750 °C for 3 h and then rapidly cooled (using tap water), Fig. 7b shows the CS magnitudes for several composites. Similarly, due to the thermal shock experienced by the fired samples during the quick cooling processes, the CS values for all composites significantly decrease as the exposure temperature increases from 250 to 750 °C. In addition, cracks may appear later on in the fired samples. However, compared to ordinary WOPC, the drop in CS values for all composites incorporating MS-NPs is lower.

**Fig. 8:**
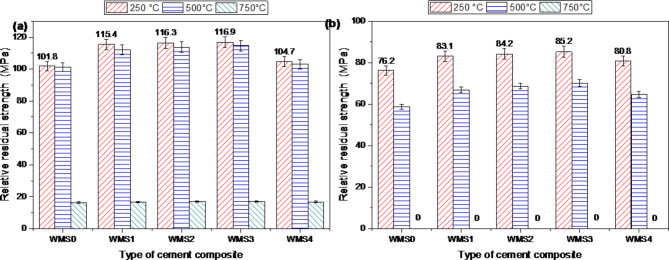
Relative residual strength for hardened composites made from WOPC-MS fired at different temperatures and cooled (**a**) gradually in air, (**b**) suddenly in water.

The relative compressive strength percentages (RCS%) for composites WOPC-MS0.25, WOPC-MS0.50, WOPC-MS0.75, and WOPC-MS1.0 that were cooled gradually in the air were, as shown in Figs. 8a and b and 115.4, 116.3, 116.9, and 104.7%, respectively, relative to plain WOPC. This suggests that 0.75 mass% MS-NPs is the ideal incorporation of MS-NPs for enhancing TD at 250 °C. This development in TD at 250 °C for OPC-0.75% MS-NPs composite paste is related to divers factors like; good dispersion of MS0.75 NPs (6.14 nm and SBET = 187m^2^/g) through the composite matrix and credible catalytic activity of MS-NPs to create a massive amount of hydration products such as (C-S-Hs I and II), C-A-S-H, C-A-H, C-F-H, CH and C-F-S-H) that precipitate in the voids of the cement matrix and generate hardened matrix that possess perfect resistance to fire decay. Obviously, upon exposure to 500 °C, the CS magnitudes were slightly reduced for all composite pastes (Mixes WMS0, WMS1, WMS2, WMS3 and WMS4). The descent in CS magnitudes is mainly attributed to the deterioration of most of the hydration products (CSHI, CSHII, CASHs, CAHs, CFH, CH, and CFSH. yet, all composite pastes containing MS-NPs have lower reduction in CS values if compared with that of plain composite. the percentages of RCS after firing at 500 °C for Mixes WMS0, WMS1, WMS2, WMS3 and WMS4 were 101.2,112.1, 113.7, 114.6 and 103.1%; supported that WOPC-MS composite pastes possess higher TD if compared with that of plain (mix WMS0).

The massive reduction in CS magnitudes seen in all tested composites after being fired to 750 °C can be attributed to the complete thermal degradation of all binding centers. As a result, it generates many fractures inside the composite matrix. The percentages of RCS at 750 °C for mixes WMS1, WMS2, WMS3 and WMS4 were 16.6, 16.9, 17.0 and 16.7%; supported that WOPC-MS composite pastes possess higher TD if compared with that of plain (mix WMS0) at high temperature. Mix WMS3 (WOPC-0.75% MS-NPs composite) acquires the highest compression magnitudes and the effective TD during treating with thermally temperature up to 750 °C, if it compared with plain WOPC.

The previously reported results exposed that all the hardened composites modified with diverse amounts of MS-NPs offered perfect TD (fire impedance) compared to other composites (plain WOPC and WOPC-MS), specially (WOPC-0.75 MS-NPs), which show the opportunity to utilize of these composites in fire impedance application.

### Phase composition

#### Differential Thermo-gravimetric analysis (TGA/DTG)

**Fig. 9:**
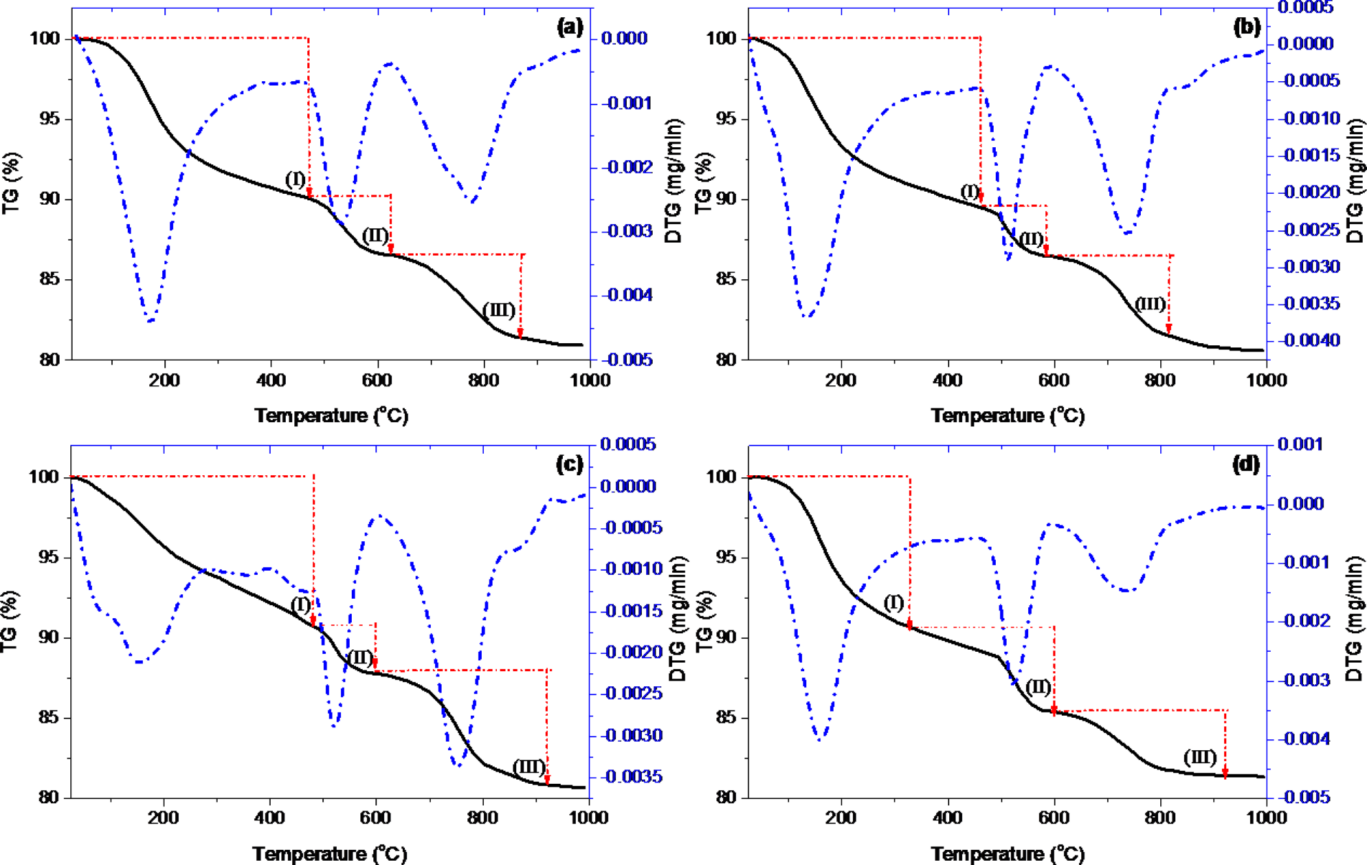
TG/DTG thermograms of hardened neat WOPC pastes (Mix WMS0) at (**a**) 7 days, (**c**) 28 days of hydration and hardened WOPC-MS pastes containing 0.75% MS (Mix WMS3) at (**b**) 7 days and (**d**) 28 days of hydration.

In order to thoroughly analyze the thermal stability of the hardened neat WOPC and hardened WOPC-MS pastes with 0.75% MS, their thermal properties were examined after 7 and 28 days of hydration. Weight loss (TG) and first derivative of weight loss (DTG) curves for hardened WOPC in combination with and without MS-NPs are shown in Fig. 9a–d. Table 3 shows how much weight each hardened paste lost at different temperature ranges. It also shows the kinetic parameter data from the Coats-Redfern plots for each step of the decomposition process. The thermal characterization graphs presented in Fig. 9a–d clearly demonstrate that each curve comprises three distinct stages of thermal degradation;

(I) The initial phase within the temperature range of 24–386 °C results in a reduction in weight due to the evaporation of hydrated products resulting from the decomposition of CSAHs, CSHs, and CAHs. The analysis of the hydrate content in the solidified neat WOPC and WOPC-MS pastes reveals that the inclusion of MS-NPs led to a greater bonding of water molecules, mostly due to the enhanced hydrophilic properties of the MS-NPs.
(II) The second phase, occurring at temperatures between 473 and 609 °C, results from pyrolysis. It is caused by the dehydroxylation of Ca(OH)_2_. The estimated mass loss for hardened neat WOPC pastes (Mix WMS0) is 4.324%, and 4.399%, at 7 days and 28 days of hydration. For hardened WOPC-MS pastes containing 0.75% MS, the estimated mass loss is 3.668%, and 4.647% at 7 days and 28 days of hydration, respectively. The outcomes show the distinct hardened pathway that WOPC followed in the presence of MS-NPs.
(III) The third phase is the pyrolysis of free lime and carbonated segments, which ends at 869, 929, 805, and 806 °C. The weight loss for hardened neat WOPC pastes (Mix WMS0) at 7 days and 28 days of hydration and for hardened WOPC-MS pastes containing 0.75% MS at 7 days and 28 days of hydration is 5.414, 6.98, 5.635, and 3.898%, respectively. Following the combustion process, the residual weight for hardened plain WOPC pastes (Mix WMS0) at 7 days and 28 days of hydration and hardened WOPC-MS pastes containing 0.75% MS at 7 days and 28 days of hydration, respectively, equals 80.95, 80.71, 80.60, and 81.36%.

The pyrolysis mechanism and thermal stability of hardened neat WOPC and hardened WOPC-MS pastes can be approximated from these collected data. The WOPC under investigation, both with and without MS-NPs, is stable at room temperature and deteriorates progressively when heated. These rigid pastes can be thermally broken down into two stages: pyrolysis and dehydration. The dehydration operation of all hardened pastes occurred in one stage. After the water molecules are eliminated, two phases of the thermal breakdown of the dried pastes take place.

On the other hand, the DTG curve of the maximum weight loss rate (RW_loss_, %/minute) can be used in two ways to talk about the default stability for hardened pastes: The term “thermal stability” can refer to either the energy of activation (E^a^) for decomposition reactions or the temperature values at which the rate of weight loss from thermal degradation is greatest (T^max^, °C). The former is more commonly used to describe kinetic stability. The starting temperatures of thermal deterioration (T^onset^, °C) are another way to describe it. It is evident that as the hardening time was increased from 7 to 28 days, the thermal stability of the neat WOPC became less stable, based on the T^max^ of the second and third degradation stages, which may be regarded as one thermal stability factor. On the other hand, by lengthening the hardening period from 7 to 28 days, hardened WOPC-MS pastes have improved thermal stability.

Based on the kinetic data in Table 3, the activation energies for the initial decomposition reaction (I) in hardened pastes are arranged in the following order: Mix WMS3-7d < Mix WMS0-28d < Mix WMS3-28d < Mix WMS0-7d. Furthermore, the order of thermal stability between the stages of thermal degradation can be described as follows: II > III > I for hardened pastes of Mix WMS0-7d, Mix WMS0-28d, and Mix WMS3-7d. The combustion stage of the hardened WOPC-MS pastes, on the other hand, exhibits the maximum activation energy after 28 days of hydration, indicating that more energy is needed to start the thermal decomposition process at the specified T^max^. The activation energy and the enthalpy of activation follow the same order: WMS3-28d > mix WMS0-7d > WMS3-7d > WMS0-28d. In contrast, the activation entropies are in the following order: mix WMS3-28d < mix WMS0-7d < mix WMS3-7d < mix < WMS0-28d. This indicates that by adding MS-NPs to the WOPC paste, the activation entropy decreased and eventually reached a minimum after 28 days of dehydration.

**Table 3:** Thermo-analytical data of hardened neat WOPC and hardened WOPC-MS pastes containing 0.75% MS after 7 and 28 days of hydration.

	Stage	Temp. range, °C	T^onset^, °C	T^max^, °C	W_loss_, %	Rw_1000_, °C	*R* ^2^	A, 1/s	E^a^, kJ/mol	∆H^a^, kJ/mol	∆S^a^, J/mol.K	∆G^a^, kJ/mol
Mix WMS0-7d	I	32–380	99	175	9.087	80.95	0.93	10.60	25.23	21.50	-228.725	123.97
	II	400–609	463	526	4.324		0.98	2.94 × 10^10^	183.03	176.38	-52.7186	218.50
	III	629–879	629	775	5.414		0.99	1.21 × 10^07^	171.40	162.69	-119.772	288.21
									∑ 379.66	∑ 360.57	∑ -401.22	∑ 630.69
Mix WMS3-7d	I	24–386	100	127	8.87	80.60	0.93	2.99	19.67	16.36	-238.297	111.68
	II	472–576	472	509	3.668		0.98	2.61 × 10^13^	221.74	215.23	3.932746	212.16
	III	576–805	618	737	5.635		0.99	2.57 × 10^08^	188.80	180.40	-94.0709	275.41
									∑ 430.22	∑ 412.00	∑ -328.44	∑ 599.25
Mix WMS0-28d	I	24–284	98	150	5.776	80.71	0.91	5.70	20.59	17.07	-233.408	115.80
	II	492–596	483	520	4.399		0.99	1.15 × 10^12^	204.56	197.97	-22.187	215.56
	III	596–929	608	753	6.98		0.98	271578.4	141.10	132.57	-151.196	287.69
									∑ 366.25	∑ 347.61	∑ -406.79	∑ 619.06
Mix WMS3-28d	I	24–369	100	157	9.734	81.36	0.97	9.37	24.33	20.76	-229.406	119.40
	II	473–577	469	518	4.647		0.97	1.41 × 10^13^	219.98	213.40	-1.2992	214.43
	III	577–806	601	744	3.898		0.97	4.86 × 10^16^	353.53	345.08	64.34119	279.64
									∑ 597.84	∑ 579.23	∑ -166.36	∑ 613.47

#### X-ray diffraction (XRD)

**Fig. 10:**
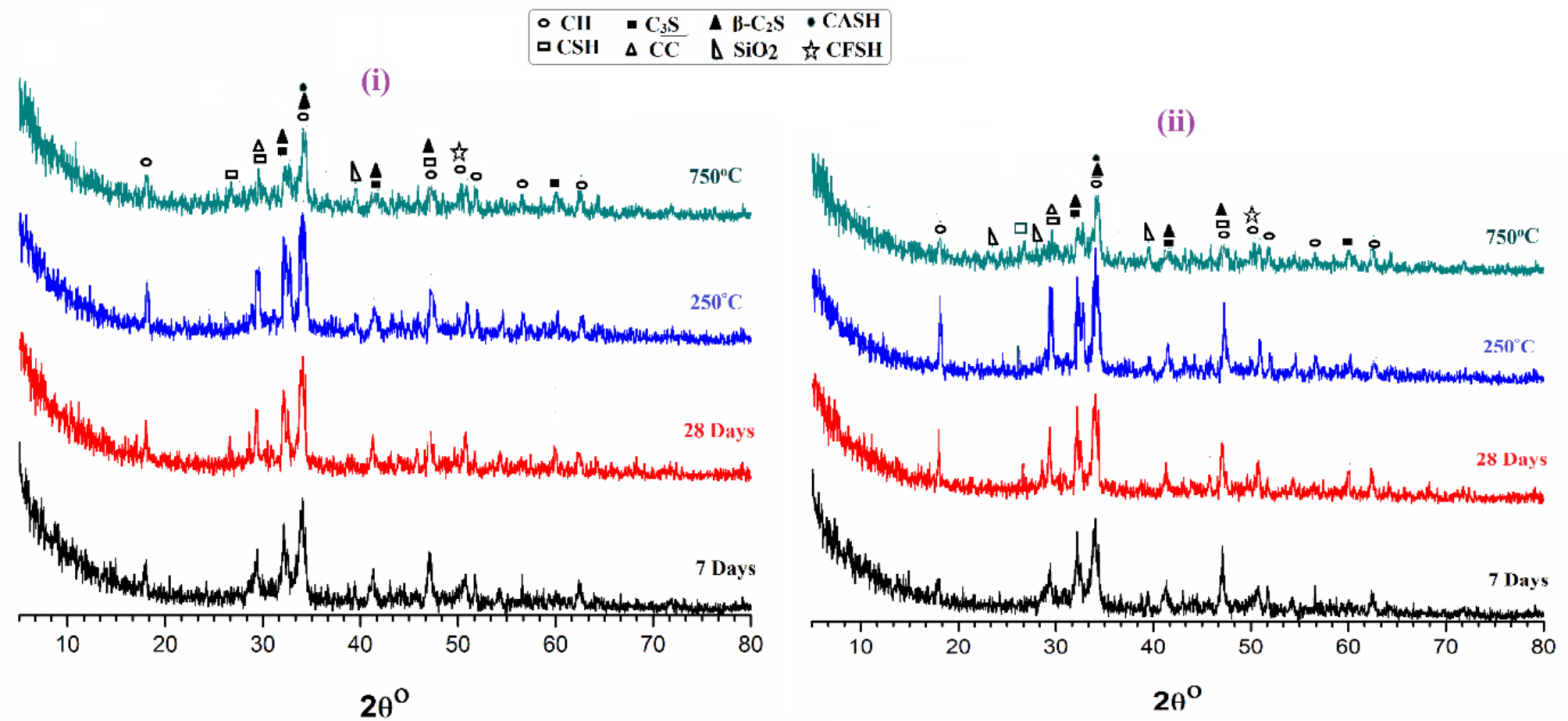
XRD-patterns for (i) hardened neat WOPC pastes (Mix WMS0) and (ii) hardened WOPC-MS pastes containing 0.75% MS (Mix WMS3).

Figure 10 (i) presents the XRD patterns of composites consisting of plain WOPC at different time intervals (7 and 28 days) and after being subjected to different thermal temperatures (250 and 750 °C) after 28 days of hydration. The diagram displays distinct peaks representing various hydration yields, including C-S-H and C-H, the primary hydration products. Additionally, there are indicative peaks of some unreacted portions of β-C_2_S and C_3_S. Furthermore, the peaks featured for the calcite (calcium carbonate; CaCO_3_) at nearly 2Ɵ of 29.32^[o 65^ are the results of the reaction of CO_2_ gas with the formed lime (CH). In addition to crystalline silica (quartz) is detected^66^. XRD patterns of the sample fired at 250 °C shows all phases mentioned earlier as well as a new phase of CASH formed as a result of the self (internal) autoclaving phenomena of unreacted fractions; and this result is in match with the findings of the compressive strength (CS). The XRD pattern demonstrates a noticeable decrease in the intensity of the prominent peaks of CSH, CH, and CASH due to the thermal degradation of hydration products at an elevated temperature of 750 °C. Furthermore, the calcite peaks, composed of calcium carbonate (CaCO_3_), vanished at 750 °C. This disappearance occurred due to the disintegration of both calcium hydroxide (CH) and calcium carbonate (CaCO_3_), resulting in the formation of calcium oxide (CaO)^67^.

Figure 10(ii) displays the XRD patterns of composite made of WOPC-0.75% MS-NPs at diverse times intervals (7 and 28 days) and after firing at various thermal temperatures (250 and 750 °C) at 28 days of hydration. It is legibly that all above-mentioned phases are existent like CSHs (as wollastonite and Clinozoisite phases), Calcite, alite, belite, and CH. Furthermore, as the hydration progressed, the diffraction lines corresponding to CSHs and CFH phases increased with hydration age up to 28 days^68,69^.

In the Fig. 10(ii) as a result of the catalytic activity of 0.75% MS-NPs, new peaks appeared corresponding to extra phases are seen such as the peaks of CASHs phase and ilavite (CFSH). In addition, the calcite and CH peaks were not as strong because of the pozzolanic interaction of 0.75% MS-NPs with CH. At a temperature of 250 °C, there was a significant rise in the intensity of CSH, CH, and CASH peaks in the specimen. This increase can be attributed to the internal reaction (self-autoclaving) of unreacted phases, namely alite and belite. Whereas, for the specimen exposed for 750 °C, a significant decline in the intensity of CSH, CH, CFSH and CASH peaks has been detected as a result of the thermal decadence of such hydration yields^69^.

### Textural and morphological features

**Fig. 11:**
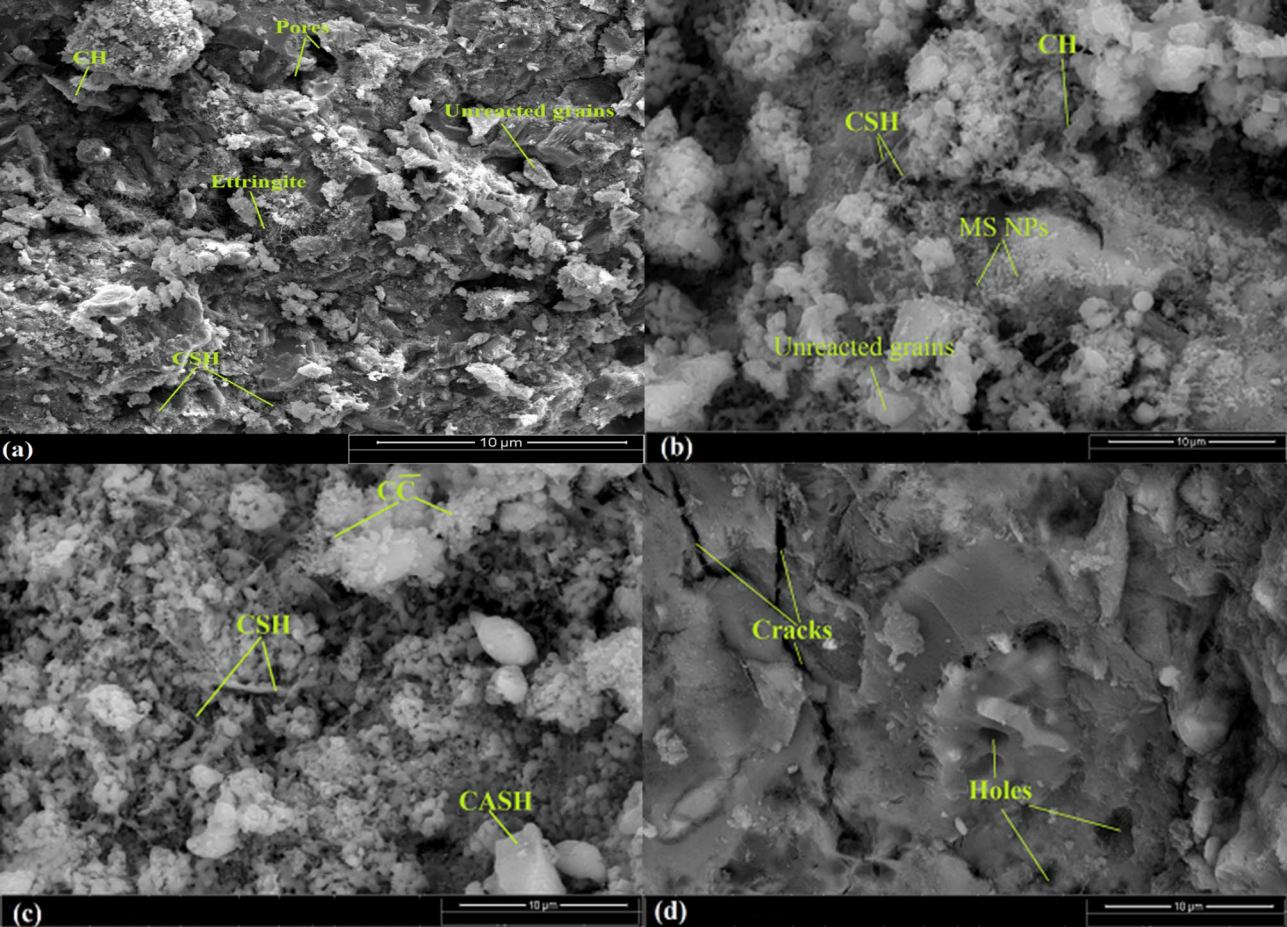
SEM images of WOPC-hardened cement pastes (Mix WMS0) at 7 days (**a**); at 28 days (**b**); after firing at 250 °C (**c**); and after firing at 750 °C (**d**).

Figure 11a and b display the microstructure and morphology of the hardened, neat WOPC pastes following 7 and 28 days of hydration. The SEM micrograph after 7 days reveal the formation of small amounts of (C-S-H; tobermorite-like) along with the creation of small hexagonal portlandite (CH) crystals. In addition to a considerable amount of unreacted particles can be well detected as shown in Fig. 11a^39^. A dense matrix was acquired after 28 days of curing time as clear in Fig. 11b, composed of massive amounts of needle crystals and fibrous C-S-H combined with portlandite (CH) which appear as accumulated hexagonal crystals. also, as a result of the side reaction of atmospheric CO_2_ gas with CH created through handling of the specimens, a tiny amount of calcite (CaCO_3_) as cubic plates crystals can be observed in the cementitious matrix. Moreover, the presence of vacuum in the cementitious matrix for the sedimentation of other new phases of hydration^70^. SEM images as illustrated in Fig. 11c for thermally treated WOPC pastes at 250 °C after 28 days of hydration shows the presence of a compact structure in the form of sheets, ill-crystallized of (CSH) and plates of CASHs that overlapped with the hexagonal plates of portlandite (CH) liberated from WOPC hydration. The attained microstructure at 250 °C affirm the internal (self-autoclaving) reaction of unreacted WOPC particles which cause of the generation of more amount of hydration phases like (C-S-H, C-H, C-A-H, C-A-S-H), forming a denser structure^4^. Figure 11d indicates SEM images of thermally treated WOPC pastes at 750 °C after 28 days of hydration showing a whole thermal degradation for almost hydration yields with different micro-cracks appearing^43^.

**Fig. 12:**
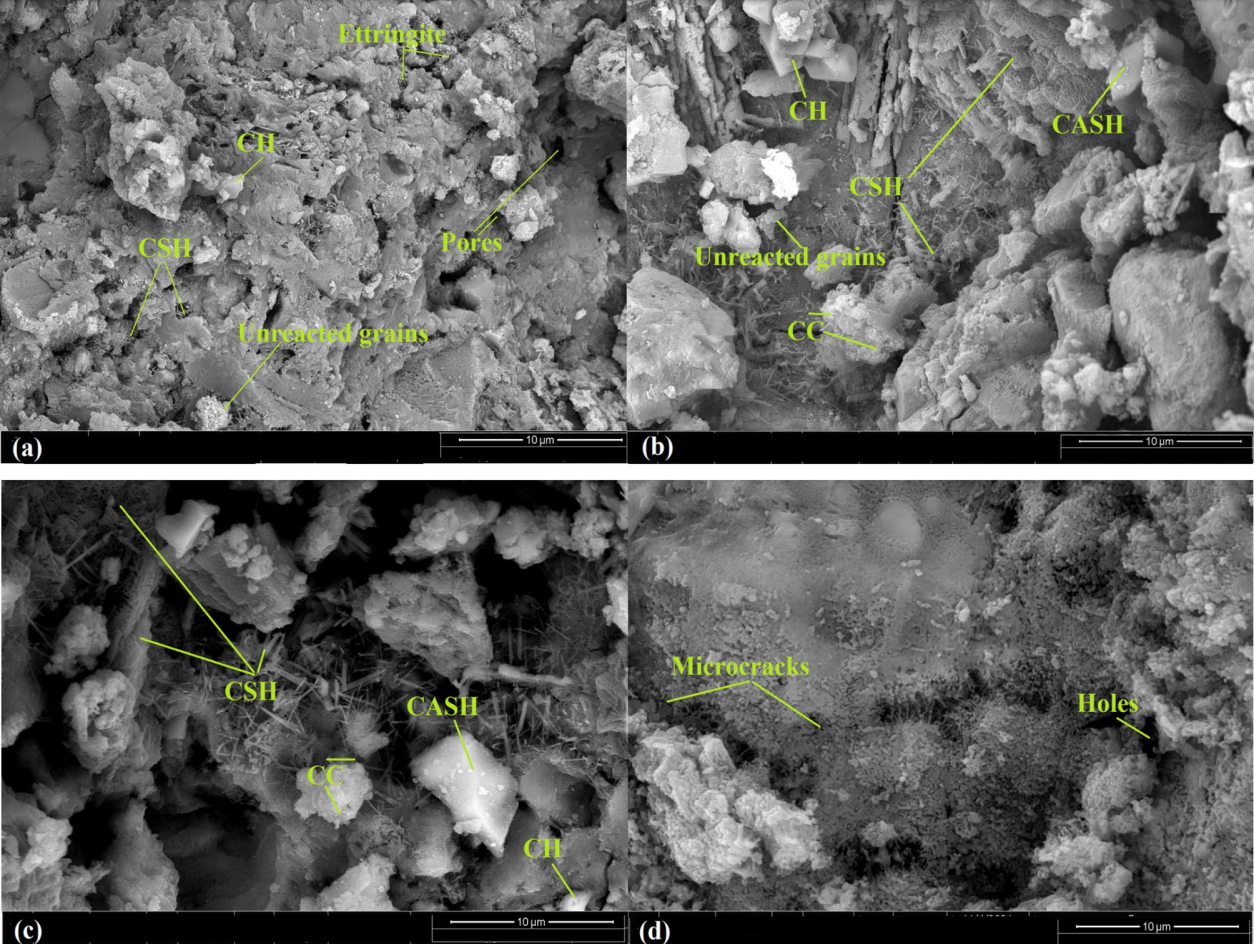
SEM images of WOPC- MS hardened cement pastes (Mix WMS3) at 7 days (**a**); at 28 days (**b**); after firing at 250 °C (**c**); and after firing at 750 °C(**d**).

Figure 12a, b clarifies the SEM images of hardened composite containing WOPC-0.75 MS-NPs (mix WMS3) after 7 and 28 days, respectively. After 7 days, the results of the SEM micrographs of this composite are less dense and include less quantity of hydration yields and a large fraction of unreacted clinker parts if compared to their SEM micrographs after 28 days of curing age. Moreover, the hardened composite containing WOPC-0.75 MS-NPs still boost of the mechanical and microstructure properties if compared with that of blank WOPC. Figure 12b, illustrates the presence of excessive hydration products as micro-rods and crystals of fibrous CSH, CASHs plates, CFH fine crystals and AFt fibers (3CaO.Al_2_O_3_.3CaSO_4_.32H_2_O); each of them are interweaved with hexagonal CH crystals. These bulk phases are responsible for the acquired hard and dense matrix for WOPC-0.75 MS-NP and this is related to catalytic activity and the pore-filling effect of MS.

SEM images of thermally treated WOPC-0.75 MS-NPs (mix WMS3) pastes at 250 °C after 28 days of hydration are illustrated in Fig. 12c. The SEM micrographs reflect a very compact structure if compared to that of a cement blank (Mix MS0) under the same firing conditions. The micrographs of this composite confirm the presence of an excessive amount of hydration products like amorphous fibrous CSH and plates of CAHs, CASHs beside CFSH fine crystals interweaved with a few hexagonal CH plates resulting in a high density matrix with minimal porosity, which is ascribed to the internal (self) autoclaving reaction which favorably affects the mechanical characteristics.

after exposure to 750 °C, Fig. 12d revealed the existence of various micro-cracks and the relatively not fully thermal ruptures for nearly all created phases as a result of still exists small quantity of fibrous CSH which may be ascribed to presence of a high alkaline content in WOPC^31^.

## Conclusion

Positive effect of eco-friendly MS-NPs on the mechanical, physical, fire resistivity, and microstructure characteristics of WOPC pastes is reported in this article. Using eco-friendly MS-NPs was successful due to essential factors such as amorphousity, nucleating side effects on WOPC, and waste recycling. This type of WOPC-MS hardened composite has high physico-mechanical properties, low CO_2_ emission, and low cost, and it is required in construction projects. The primary influential factors in the performance of WOPC were amorphous silicate, color, and particle size.

**The results of this investigation can be summarized as follows: -**.

1. The results obtained from the experiments proved the suitability of recycled agricultural waste for improving the properties of WOPC pastes for the preparation of construction materials with outstanding durability and mechanical characteristics as well as fire resistivity.
2. Inclusion of mesoporous silica SiO_2_ nanoparticles (0.75 mass %) in WOPC pastes motivate the thermal stability.
3. The composite WMS3 (WOPC–0.75 MS) provides numerous advantages from both an economic and environmental perspective. Among all the studied nanocomposites, this mix had the greatest CS values, the shortest setting time compared to plain, and the highest RS% after firing, making it the best choice for general construction purposes.
4. The massive types of hydration products in the WOPC matrix were affirmed via SEM, XRD, and TG/DTG techniques, especially after a 0.75% addition of MS-NP.

## Data Availability

Availability of data and materials:-All data generated or analyzed during this study are available upon request from all authors of this paper.
